# Difference distance map data of alternative crystal forms of UlaA

**DOI:** 10.1016/j.dib.2016.11.087

**Published:** 2016-12-03

**Authors:** Ake Vastermark, Adelle Driker, Jingwei Weng, Xiaochun Li, Jiawei Wang, Milton H. Saier

**Affiliations:** a Department of Molecular Biology, University of California at San Diego, La Jolla, CA 92093-0116, USA; b Shanghai Key Laboratory of Molecular Catalysis and Innovative Materials, Department of Chemistry and Institute of Biomedical Sciences, Fudan University, Shanghai, People’s Republic of China; c Laboratory of Cell Biology, Howard Hughes Medical Institute, The Rockefeller University, New York, New York 10065, USA; d State Key Laboratory of Biomembrane and Membrane Biotechnology, School of Life Sciences, Tsinghua University, Beijing, People’s Republic of China

**Keywords:** C2A, P2_1_A, Space group names, APC, Amino acid-Polyamine organoCation superfamily of secondary carriers, PTS, Bacterial Phospho-Transferase System, NMA, Normal Mode Analysis

## Abstract

We introduce the value of information obtained by comparing alternative crystal forms of the same sub-state (of outward open UlaA, our example protein), which is found in the same lattice configuration but different space groups. We compare instability estimates obtained using this new method (alternative crystal forms) with temperature factors. Using a transport assay result, we correlate observations for two homologous secondary structure elements, and show that the alternative states method for obtaining instability estimates provide differentiating information about an important and immobilized mid-TMS region. The data presented in this article are related to the article entitled “The V-motifs facilitate the substrate capturing step of the PTS elevator mechanism” (A. Vastermark, A. Driker, J. Weng, X. Li, J. Wang, M.H. Saier Jr., 2016).

**Specifications Table**

**Table:** 

Subject area	*Physics*
More specific subject area	*Biophysics, bioinformatics*
Type of data	*3 tables, 3 molecular graphics, 1 alignment, 1 graph, 1 schematic figure, 5 distance maps*
How data was acquired	*Secondary analyses of published data*
Data format	*Raw (*Figs. 2, 4, 9–12, Tables 2-3*), Analyzed (*Fig 1, 3, 5–8, Table 1*)*
Experimental factors	*Secondary analyses of published data, distance maps*
Experimental features	*Computational analysis*
Data source location	*The data are accessible within the article*
Data accessibility	*The data are accessible within the article*

**Value of the data**

• Alternative crystal forms of outward open UlaA structure is valuable to interpret MD simulation of MalT substrate catching step, constituting 25% of the “elevator mechanism” of the PTS group translocation process.
• The strength of the alt. crystal data might be that it represents two real low energy collective states [1] that the protein prefers, in the crystal hindering environment.

## 1 Data

○ **Fig. 1.** Mapping between secondary structural elements.
○ **Fig. 2.** Delta distance map, showing 4RP8.C-4RP9 (outward occluded (P2_1_B)-outward open (C2A)).
○ **Fig. 3, A–B.** Superimposed side view of half space filling Michael Sanner׳s molecular surface of the substrate binding space in the C2A (blue) and P2_1_A (yellow) states (panel A). Hydrogen bonds coordinate vitamin C in the two conformations (panel B).
○ **Fig. 4, A–B.** Variable appearance of Log_10_-transformed B (temperature) factor of C2A (red and yellow lines) and P2_1_A (blue line) crystal forms in the vicinity of pivot residues Gly58 (panel A) and Gly286 (panel B).
○ **Fig. 5, A–B.** The side chains coordinating vitamin C are primarily attached to secondary structural elements of the immobile core domains (panel A). They display limited macro-movement during the C2A →P2_1_A comparison (panel B).
○ **Fig. 6, A–B.** Salt bridges in the C2A and P2_1_A crystal forms (A and B) of UlaA.
○ **Fig. 7, A–B.** Binary alignment of UlaA and MalT (panel A), and binary alignment of TMS7 region (panel B) of UlaA (sequence A) and MalT (sequence B).
○ **Fig. 8.** Nearby (5 Å) atoms of CA of G286 of UlaA (4RP9).
○ **Fig. 9, A–D (cartoonwise).** Triplicate 1 (“Rep 1”) of MD simulation of MalT substrate release by TMS7 mechanism (outward open, substrate present).
○ **Fig. 10 A–D.** Triplicate 2 (“Rep 2”) of MD simulation of MalT substrate release by TMS7 mechanism (outward open, substrate present).
○ **Fig. 11 A–D.** Triplicate 3 (“Rep 3”) of MD simulation of MalT substrate release by TMS7 mechanism (outward open, substrate present).
○ **Fig. 12 A–C**. Δ-distance maps representing the difference between the outward open C2A and P2_1_A crystal forms of UlaA, using conditional formatting (panel A), ESCET (panel B), or RR distance maps (panel C).

■ **Table 1.** Tabular form of a proposed alignment of secondary structural elements of UlaA and ChbC. This alignment is not “linear” since we cannot rearrange sequential elements.
■ **Table 2.** Hydrogen bond distances in the C2A state of the Vitamin C binding site.
■ **Table 3.** Hydrogen bond distances in the P2_1_A state of the Vitamin C binding site.

## 2 Experimental design, materials and methods

### 2.1 Δ-distance maps

*Δ*-distance maps were calculated as follows. The largest common set of C_α_ atoms was identified from the PDB files of the two conformational states of UlaA. Each state was represented as a matrix of all pairwise distance measurements. Bypassing previous convention (the “inward–outward” convention), the matrix of the state (C2A) was subtracted from the matrix of the (P2_1_A) state.

The following parameters were used for ESCET normalization [2]. For 4RP8.A, *D*_min_=2.36 (resolution), *N*_par_=26604 (number of parameters used in refinement, estimated), *N*_obs_=49177 (number of reflections), *Cpl*=96.1 (completeness), *R*_free_=0.239 (free *R* value; fit to data used in refinement), and *R*_all_=0.199 (*R* value, working and test set). For 4RP9, *D*_min_=1.65, *N*_par_=26604, *N*_obs_=71471, *Cpl*=94.6, *R*_free_=0.174, and *R*_all_=0.136.

### 2.2 MD simulations of MalT

A triplicated protein-membrane system was generated with CHARMM membrane builder using 50.000 water molecules, 200-250 ns production runs in the NAMD 2.9 package. Simulations are taken from McCoy et al. MalT structure paper, see original paper for details [3].
