# Supplementary material for: Difference distance map data of alternative crystal forms of UlaA
Source: Data Brief. 2016 Dec 3;10:198–201. doi: 10.1016/j.dib.2016.11.087 (PMC5154960; doi:10.1016/j.dib.2016.11.087)
Supplement: Supplementary file 1 — Supplementary material [file mmc1.pdf]

Manuscript No.: DIB-D-16-00773

Title: Difference distance map data of alternative crystal forms of UlaA

Journal Title: Data in Brief

Corresponding Author: Ake Vastermark

All Authors: Ake Vastermark; Adelle Driker; Jingwei Weng; Xiaochun Li; Jiawei Wang; Milton H. Saier

Submit Date: Oct 06, 2016

Conflict of interest statement

The authors declare that they have no competing interests.

Yours sincerely,

Ake Vastermark

Corresponding author
