# Supplementary figures and images for: Difference distance map data of alternative crystal forms of UlaA
Source: Data Brief. 2016 Dec 3;10:198–201. doi: 10.1016/j.dib.2016.11.087 (PMC5154960; doi:10.1016/j.dib.2016.11.087)

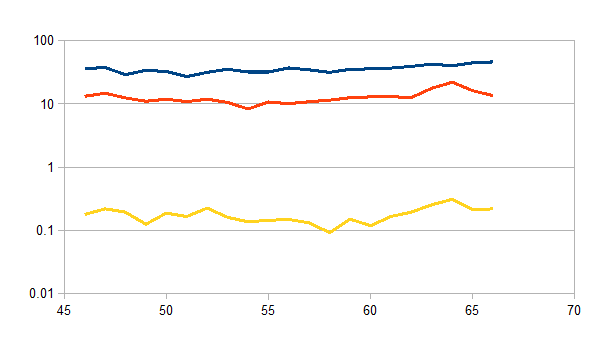

Supplement: Supplementary file 2 — Supplementary material [file mmc2.zip › 4A.png]

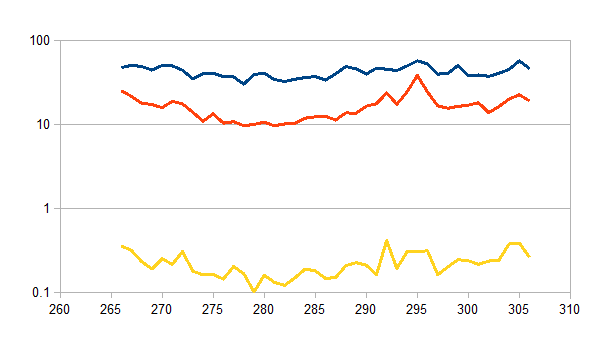

Supplement: Supplementary file 2 — Supplementary material [file mmc2.zip › 4B.png]

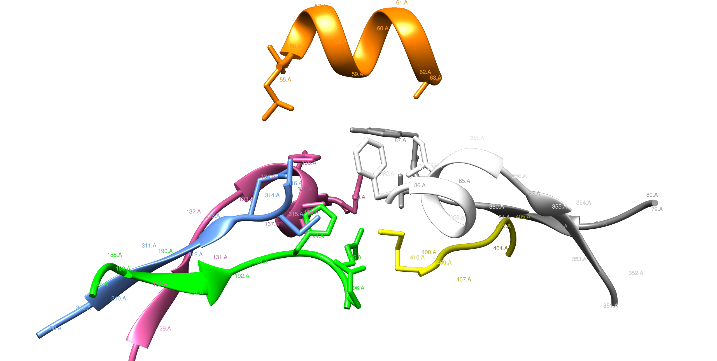

Supplement: Supplementary file 2 — Supplementary material [file mmc2.zip › 5A.png]

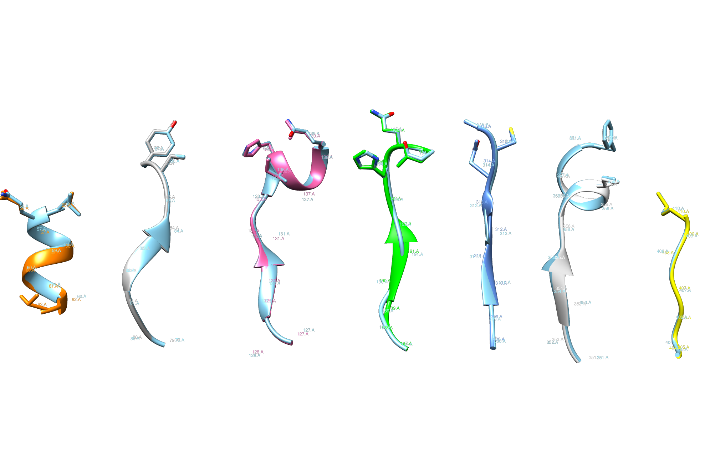

Supplement: Supplementary file 2 — Supplementary material [file mmc2.zip › 5B.png]

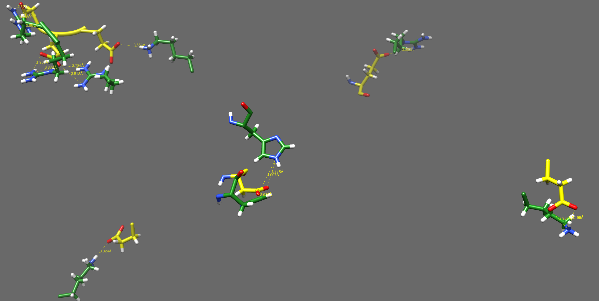

Supplement: Supplementary file 2 — Supplementary material [file mmc2.zip › 6A.png]

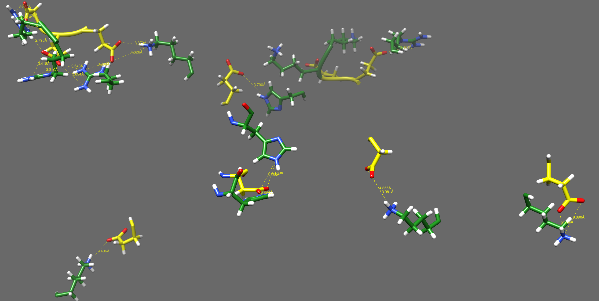

Supplement: Supplementary file 2 — Supplementary material [file mmc2.zip › 6B.png]

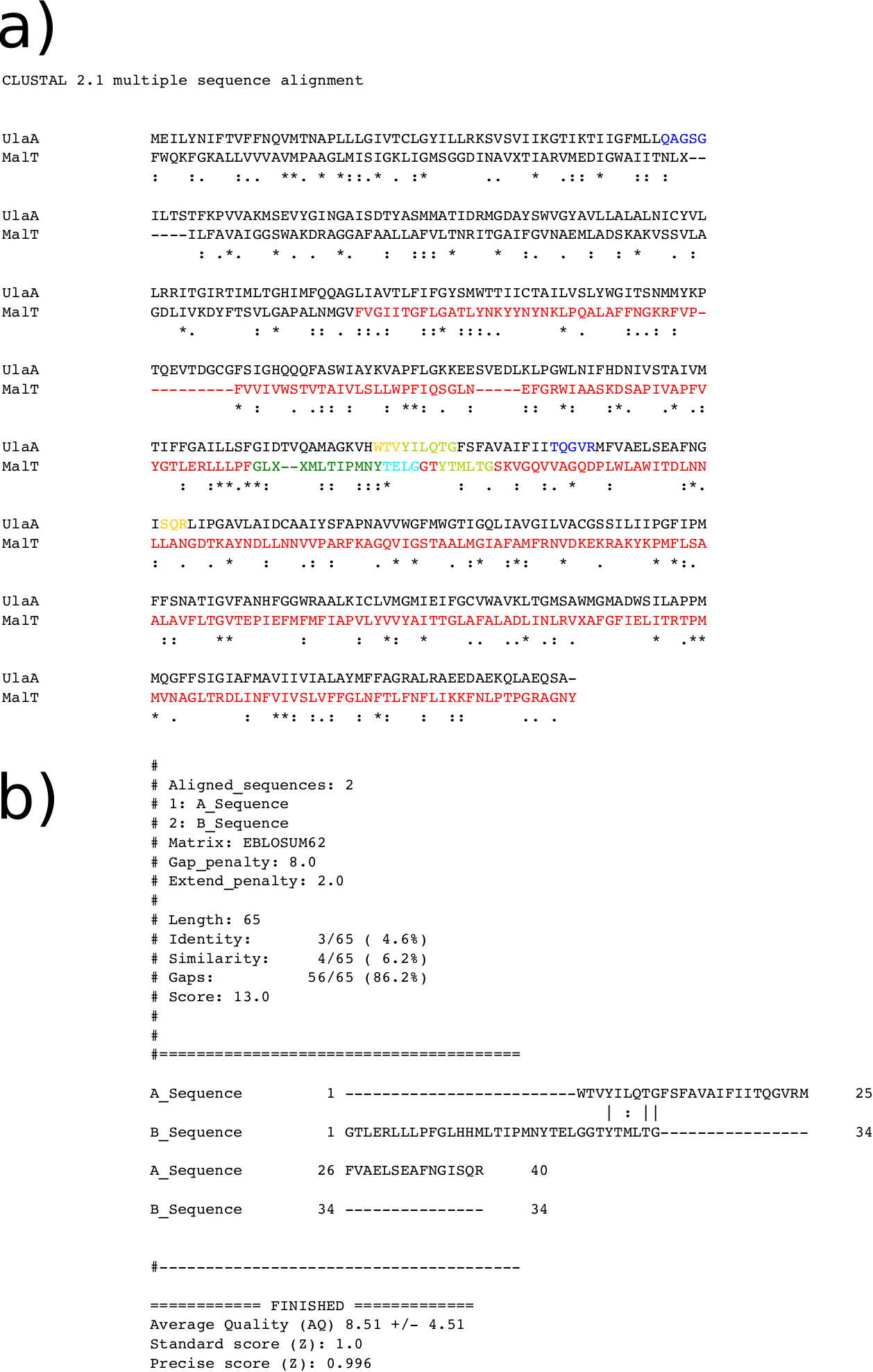

Supplement: Supplementary file 2 — Supplementary material [file mmc2.zip › 7.png]

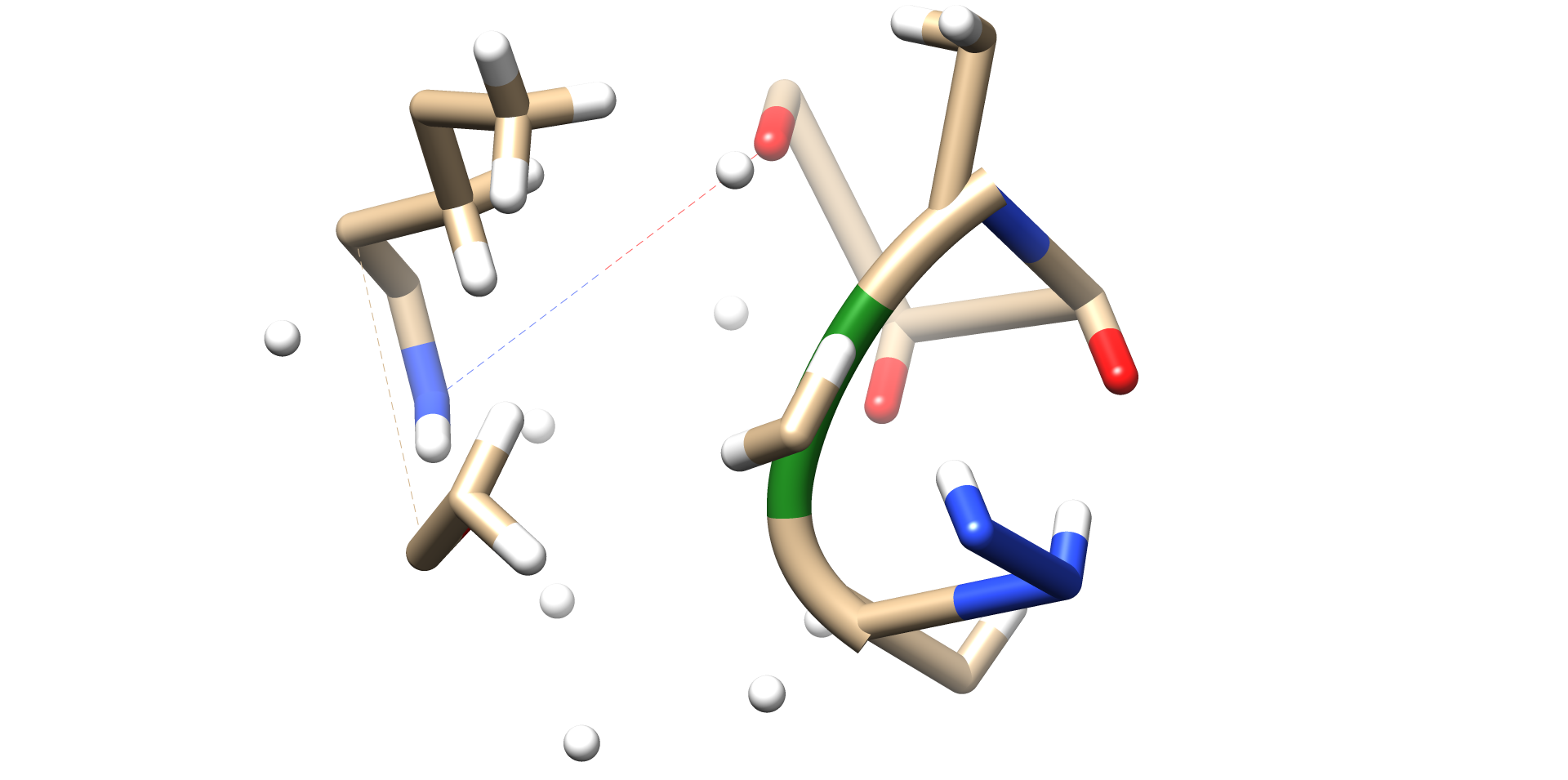

Supplement: Supplementary file 2 — Supplementary material [file mmc2.zip › 8.png]

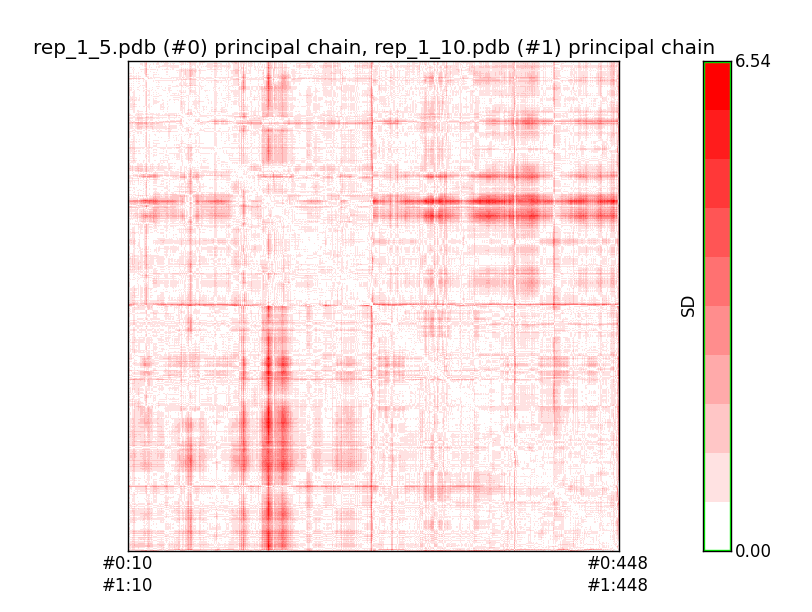

Supplement: Supplementary file 2 — Supplementary material [file mmc2.zip › 9A.png]

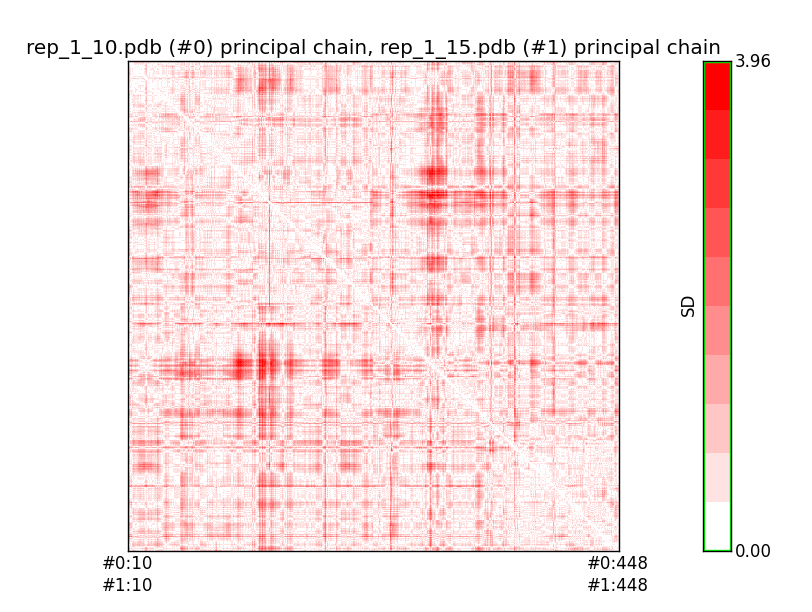

Supplement: Supplementary file 2 — Supplementary material [file mmc2.zip › 9B.png]

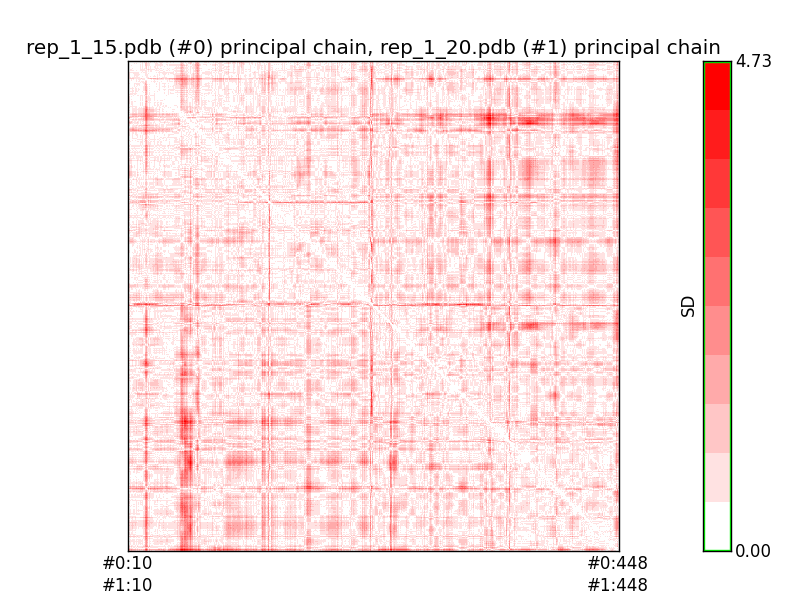

Supplement: Supplementary file 2 — Supplementary material [file mmc2.zip › 9C.png]

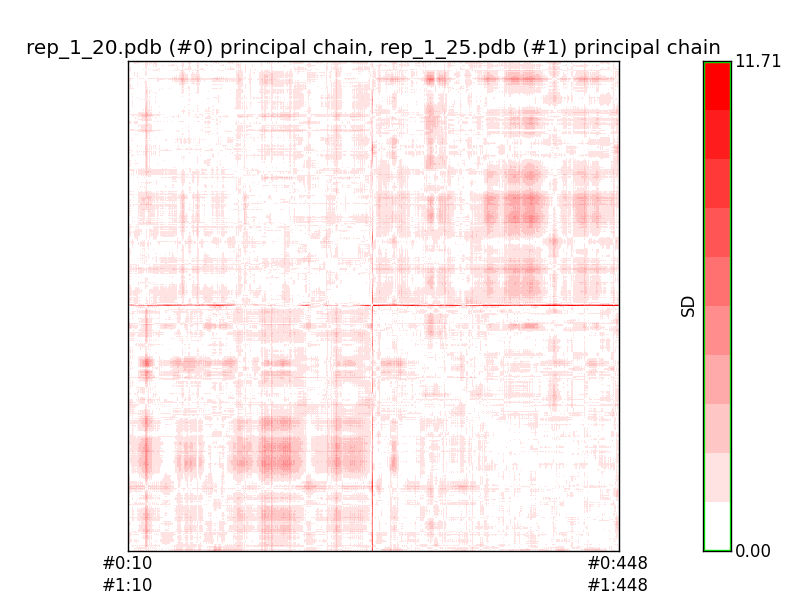

Supplement: Supplementary file 2 — Supplementary material [file mmc2.zip › 9D.png]

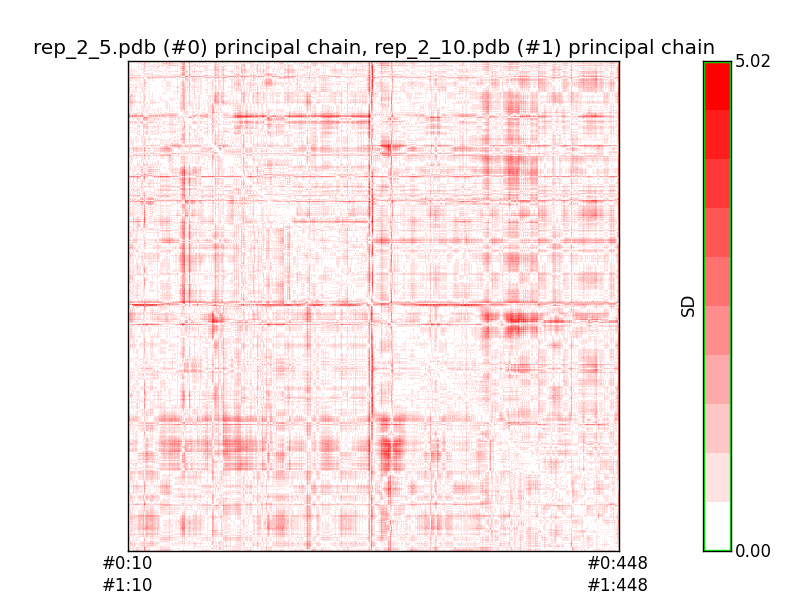

Supplement: Supplementary file 2 — Supplementary material [file mmc2.zip › 10A.png]

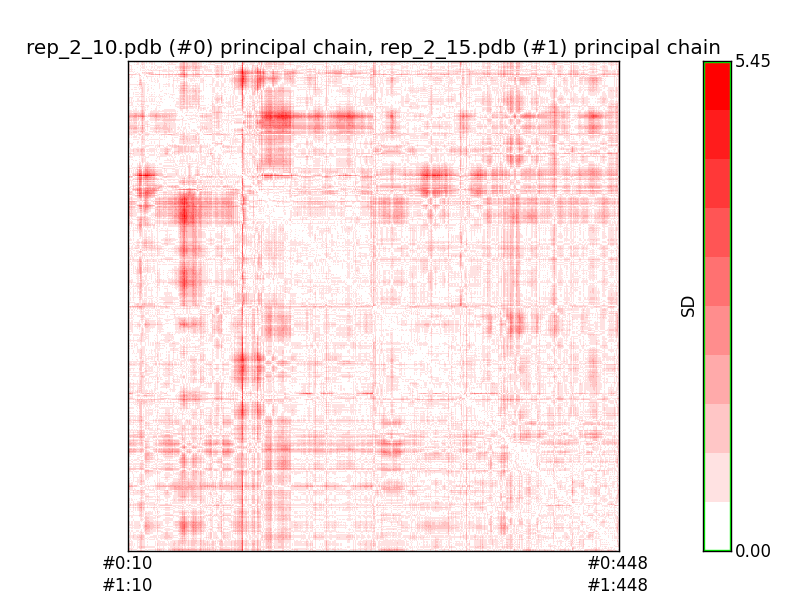

Supplement: Supplementary file 2 — Supplementary material [file mmc2.zip › 10B.png]

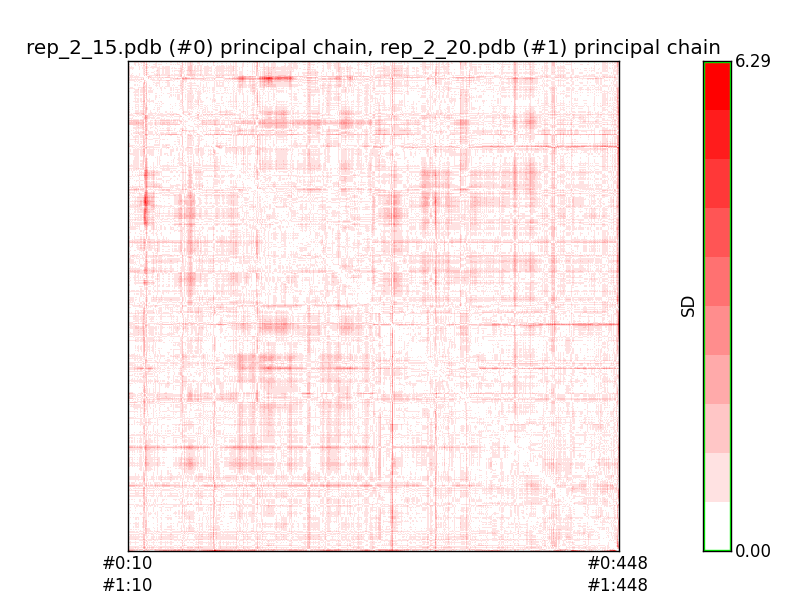

Supplement: Supplementary file 2 — Supplementary material [file mmc2.zip › 10C.png]

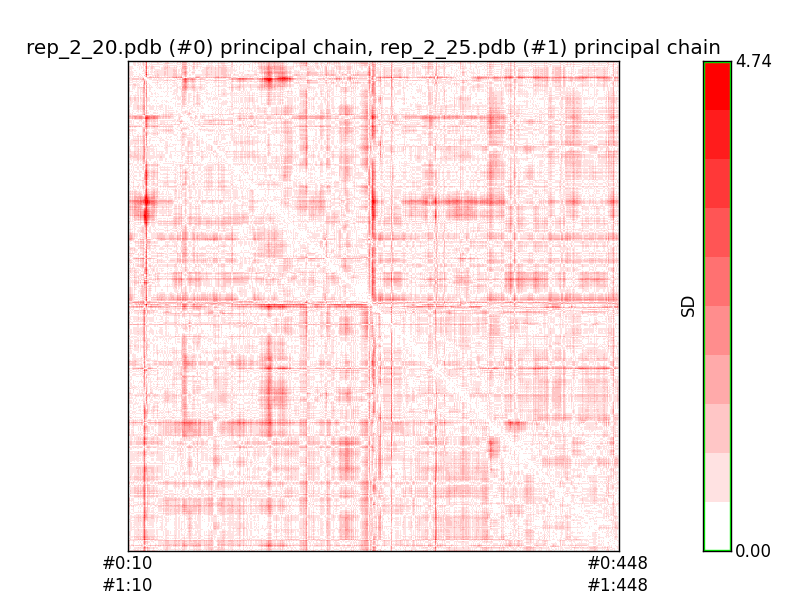

Supplement: Supplementary file 2 — Supplementary material [file mmc2.zip › 10D.png]

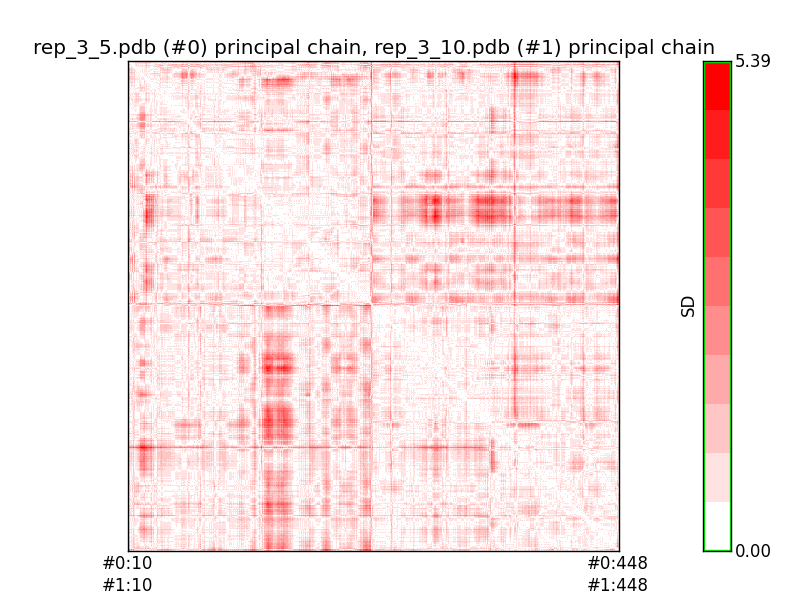

Supplement: Supplementary file 2 — Supplementary material [file mmc2.zip › 11A.png]

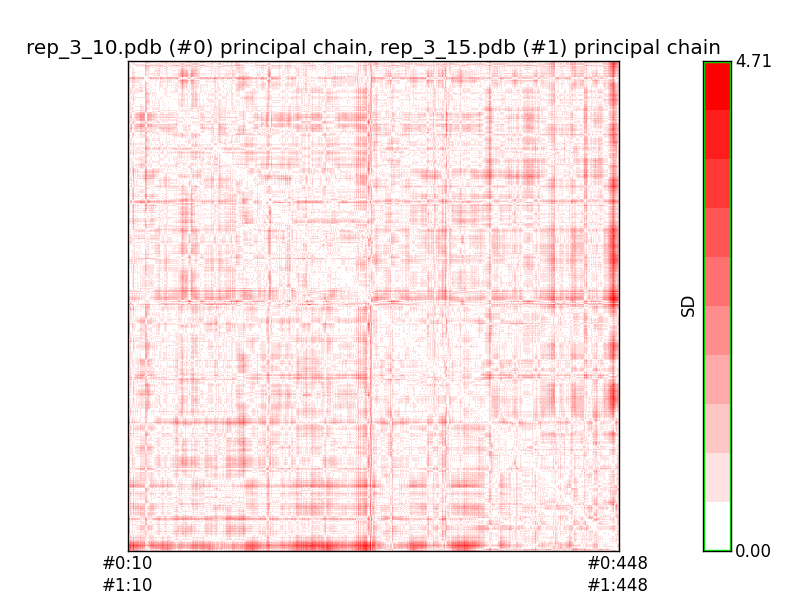

Supplement: Supplementary file 2 — Supplementary material [file mmc2.zip › 11B.png]

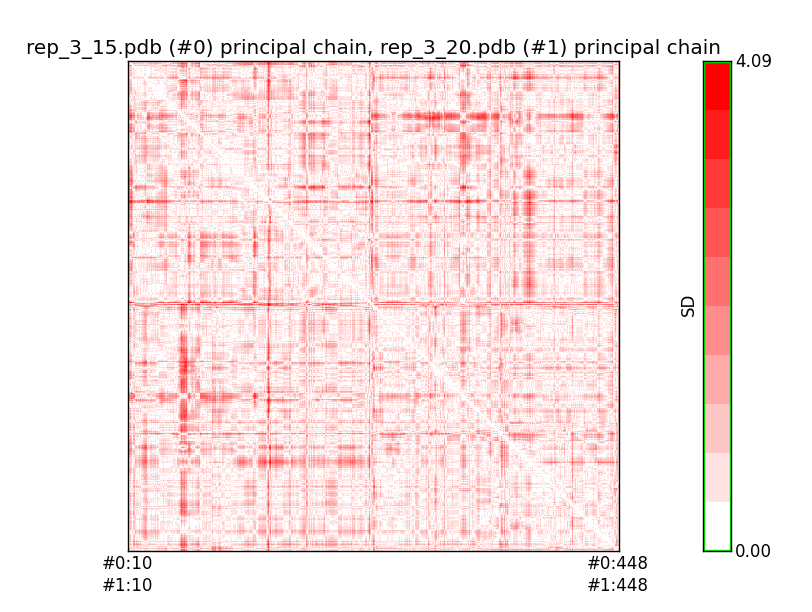

Supplement: Supplementary file 2 — Supplementary material [file mmc2.zip › 11C.png]

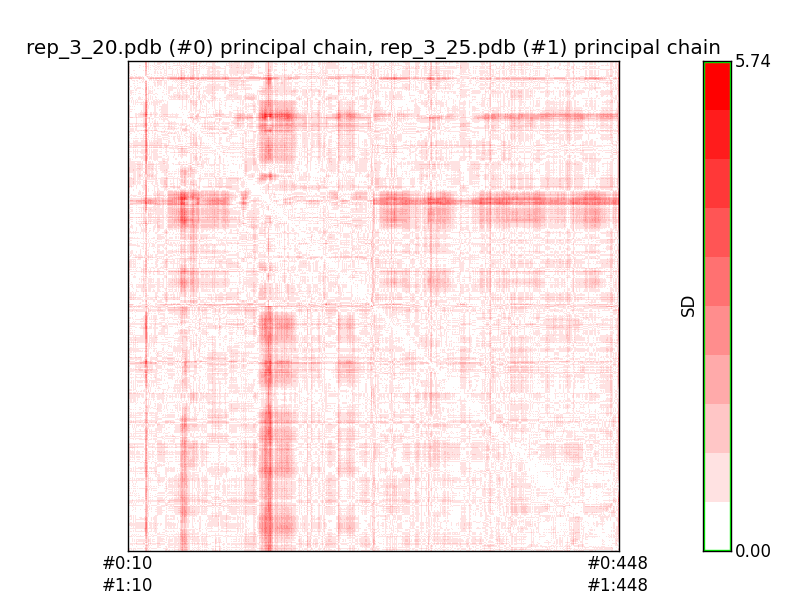

Supplement: Supplementary file 2 — Supplementary material [file mmc2.zip › 11D.png]

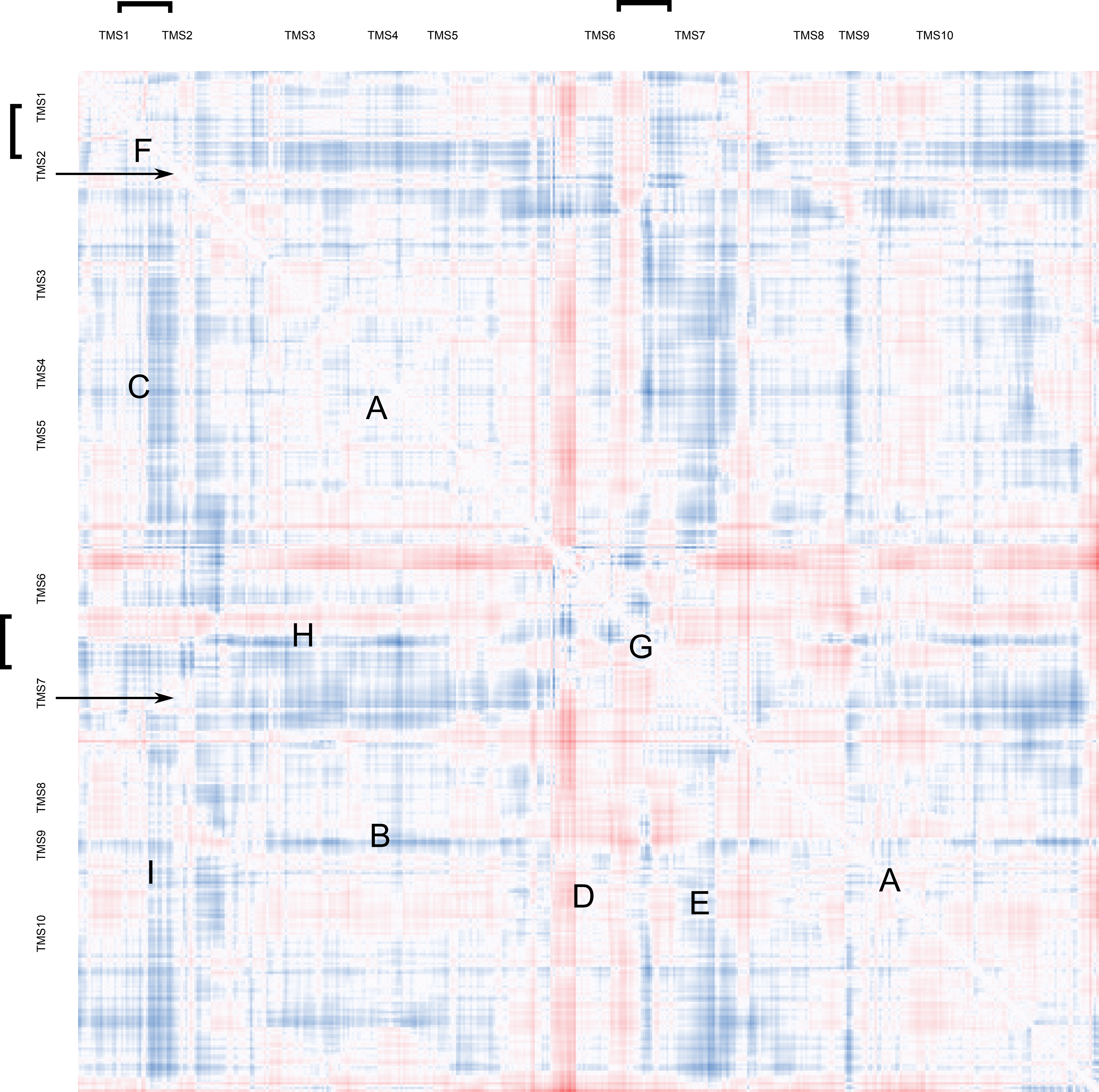

Supplement: Supplementary file 2 — Supplementary material [file mmc2.zip › 12A.pdf]

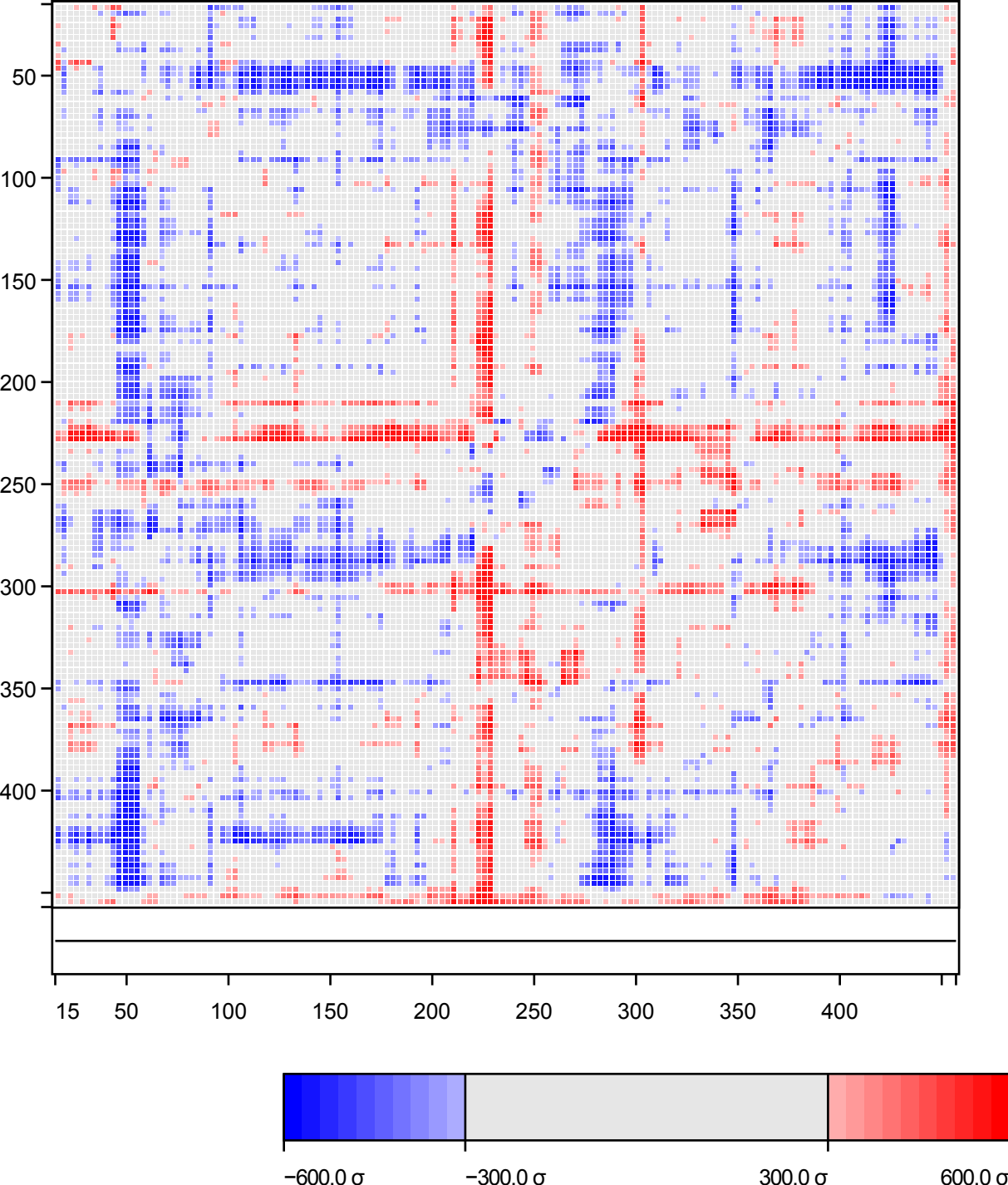

Supplement: Supplementary file 2 — Supplementary material [file mmc2.zip › 12B.pdf]

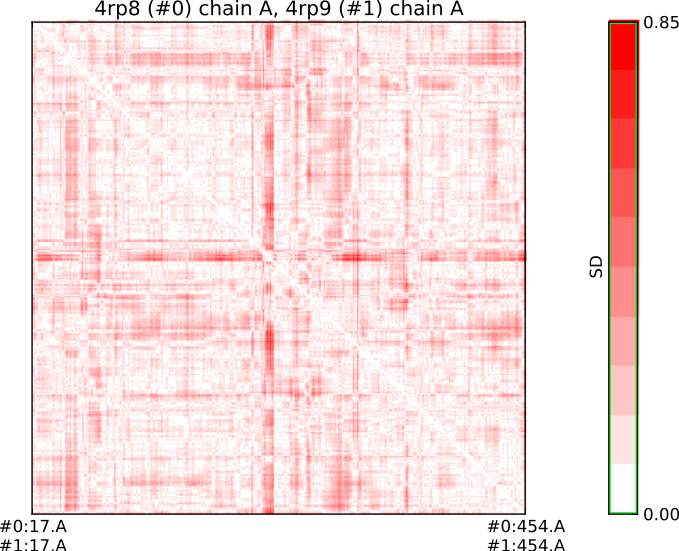

Supplement: Supplementary file 2 — Supplementary material [file mmc2.zip › 12C.tif]

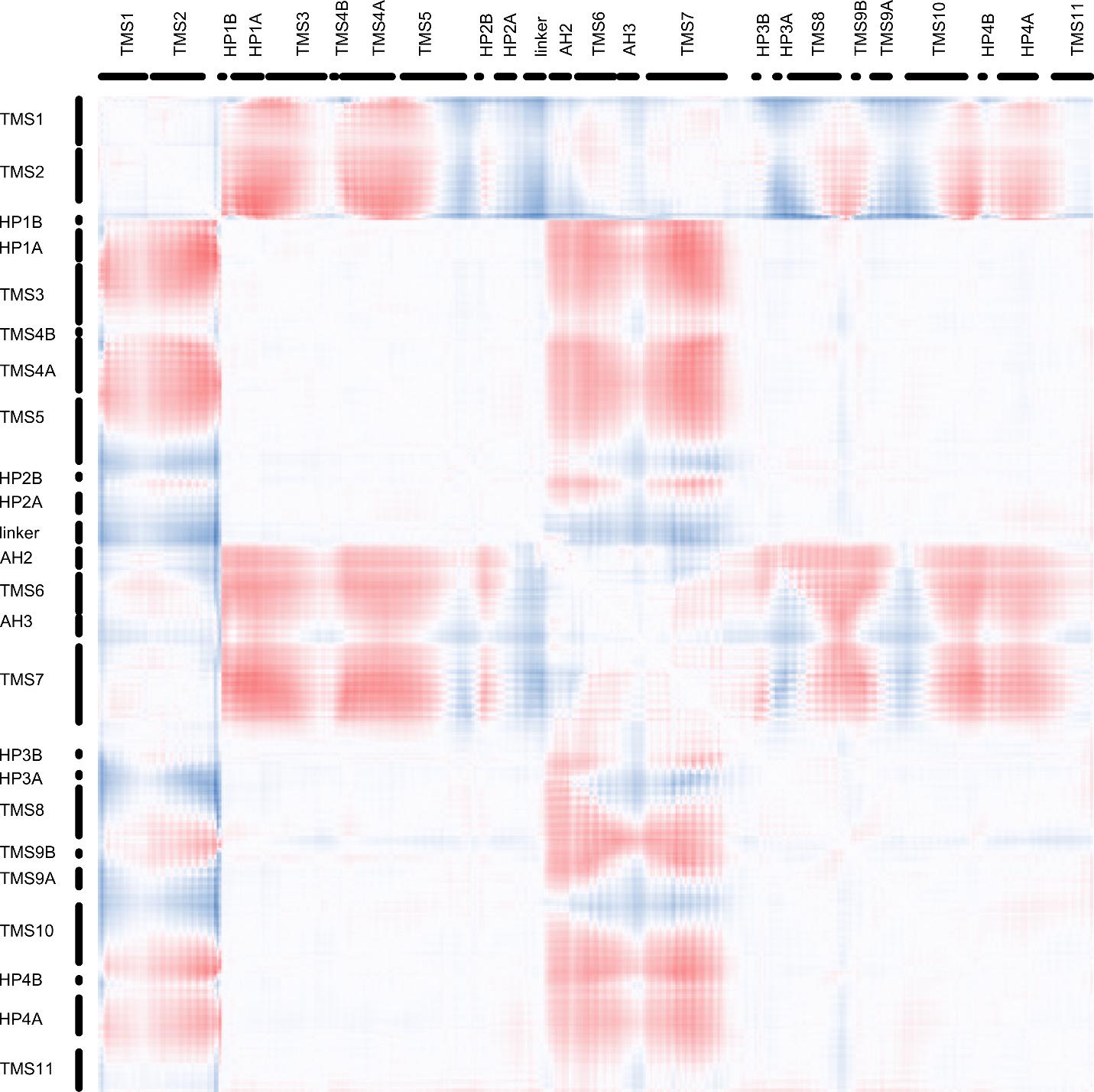

Supplement: Supplementary file 2 — Supplementary material [file mmc2.zip › 2.jpg]

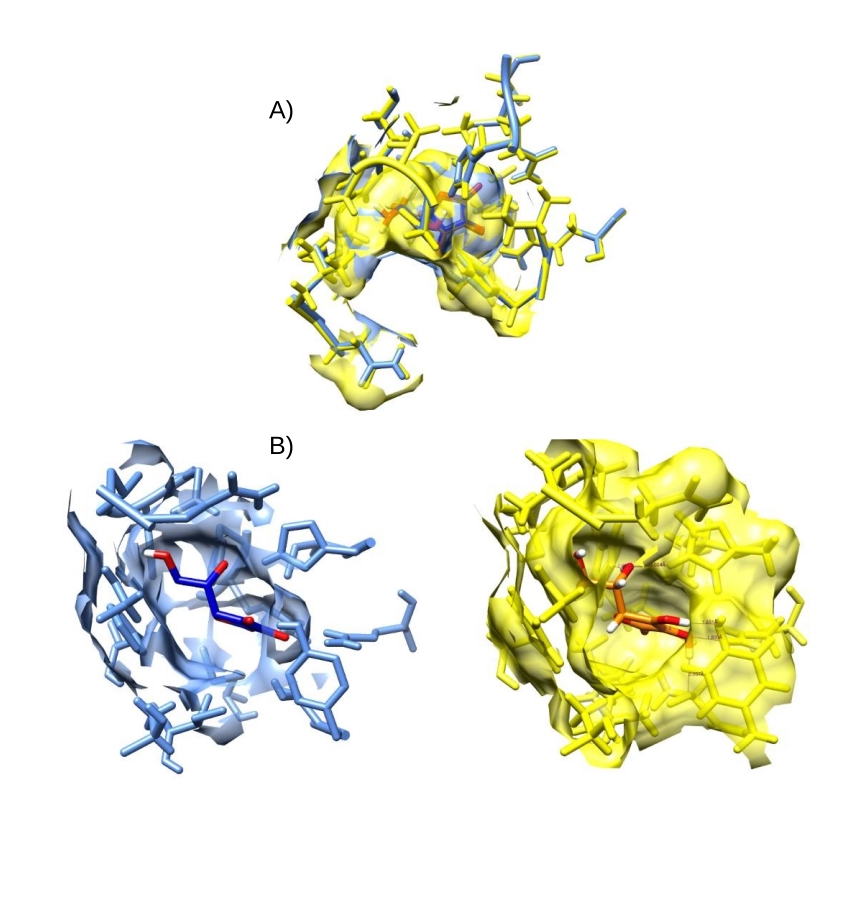

Supplement: Supplementary file 2 — Supplementary material [file mmc2.zip › _3.tiff]

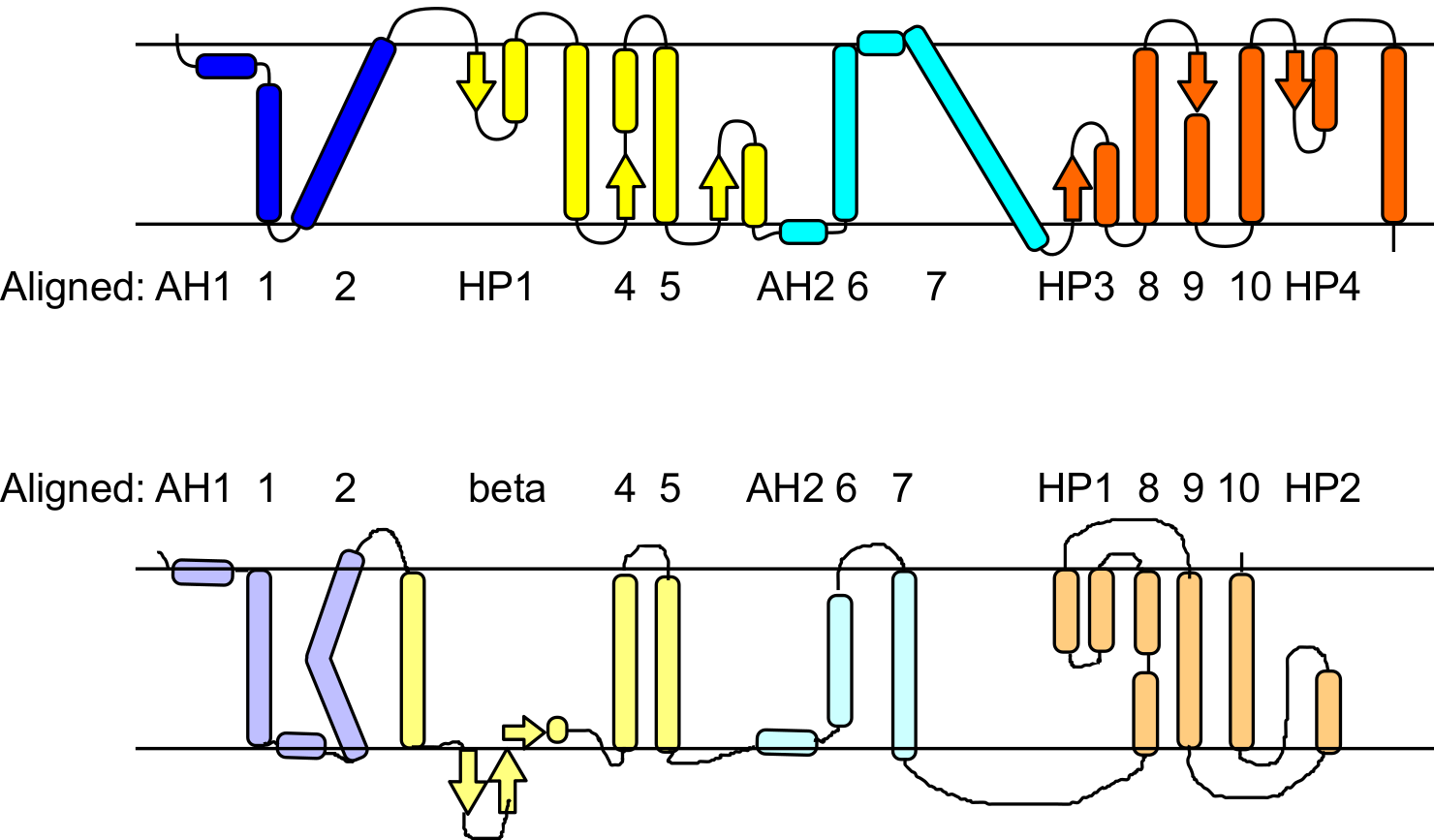

Supplement: Supplementary file 2 — Supplementary material [file mmc2.zip › _1.tiff]
