# Supplementary material for: Difference distance map data of alternative crystal forms of UlaA
Source: Data Brief. 2016 Dec 3;10:198–201. doi: 10.1016/j.dib.2016.11.087 (PMC5154960; doi:10.1016/j.dib.2016.11.087)
Supplement: Supplementary file 3 — Supplementary material [file mmc3.pdf]

Data in brief article:

Difference distance map data of alternative crystal forms of UlaA

Authors: Ake Vastermark[1,#], Adelle Driker[1], Jingwei Weng[2], Xiaochun Li[3], Jiawei Wang[4] and Milton H. Saier, Jr.[1]

Affiliations:

1. Department of Molecular Biology, University of California at San Diego, La Jolla, CA 92093-0116, USA
2. Shanghai Key Laboratory of Molecular Catalysis and Innovative Materials, Department of Chemistry and Institute of Biomedical Sciences, Fudan University, Shanghai, People's Republic of China
3. Laboratory of Cell Biology, Howard Hughes Medical Institute, The Rockefeller University, New York, New York 10065, USA
4. State Key Laboratory of Biomembrane and Membrane Biotechnology, School of Life Sciences, Tsinghua University, Beijing, People's Republic of China

Contact email: msaier@ucsd.edu (#)

Figure 1. Mapping between secondary structural elements (UlaA vs. ChbC).
This figure aligns the 10 TMSs sequentially, with the hairpins added non-sequentially. Note that both N-termini are placed at the top of the figure, even though the N-termini in UlaA are periplasmic and that in the case of ChbC, they are cytoplasmic.

Figure 2. Delta distance map, showing 4RP8.C – 4RP9 (outward occluded ($P2_1B$)-outward open (C2A)).
There is no internal movement observed inside V1, core 1, and core 2 (“rigid body-like”); there is a slight movement inside V2: during the transition to the more inward state, AH2 has separated slightly from TMS7 while TMS6 has slightly approached TMS7.
The original model predicted that core 1 and V1 approached each other while core 2 and V2 separated from each other. This prediction was verified, except for the end of core 1. Core 2 and V1 separated, except some regions: TMS2 and TMS9B, V1 and HP4. However, V2 generally approached core 2 (differing from expectation based on the model): V2 only separated from HP3 and TMS9A.

Figure 3 (A, B). Superimposed side view of half space filling Michael Sanner’s molecular surface of the substrate binding space in the C2A (blue) and $P2_1A$ (yellow) states (panel A). Hydrogen bonds coordinate vitamin C in the two conformations (panel B).
Panel A: side view. Yellow is the $P2_1A$ form. The VDW surface is more blue (patchy coverage) on the outside of the ring of ascorbate, meaning that the MSMS surface constricts the aromatic ring in the $P2_1A$ form, but this is not always true. The ring shape around the entrance to the cavity is, in fact, more constricted in the C2A (open) form, meaning that when C2A transitions to $P2_1A$, the entrance relaxes as the hydrogen bonds and VDW surface of the ascorbate ring are optimized to fit. The two differ by as little as 0.9 RMSD for the atoms shown here, does not reflect the overall shape, but the exact hydrogen bond distances. Panel B: C2A open (blue; patchy MSMS surface) and $P2_1A$ (yellow; continuous MSMS surface). Hydrogen bonds are shown for the $P2_1A$ form.

Figure 4 (A, B). Variable appearance of Log10-transformed B (temperature) factor of C2A (red and yellow lines) and P21A (blue line) crystal forms in the vicinity of pivot residues Gly58 (panel A) and Gly286 (panel B).
The yellow line is the coordinate error estimated by inversion of full LS matrix of C2A (taken from the original SHELXL log file).

Figure 5 (A, B). The side chains coordinating vitamin C are primarily attached to secondary structural elements of the immobile core domains (panel A). They display limited macro-movement during the C2A $\rightarrow P2_1A$ comparison (panel B).
Peptide strands of the UlaA binding site are composed of hairpin fragments (A). The silver colored 2˚ structural elements from left to right represent the C2A configuration, and the colored elements represent the $P2_1A$ configuration (B). From left to right: TMS2 (orange), HP1A (white), TMS4A (purple), HP2B/A (green), HP3A (cornflower blue), TM9A (grey) and HP4B/A (yellow), with their highest displayed side chain density oriented upwards. The re-entrant loops might provide suspension, effectively uncoupling 2˚ structure remodeling from the binding space (C2A $\rightarrow P2_1A$ comparison).

Figure 6 (A, B). Salt bridges in the C2A and $P2_1A$ crystal forms (A and B) of UlaA.
Salt bridges (n=16) in the C2A open state (4RP9; panel A). In the $P2_1A$ crystal state, the number of salt bridges increased to n=23, meaning that the transition from C2A to $P2_1A$ yielded energy and stabilized the $P2_1A$ conformation.

Figure 7 (A, B). Binary alignment of UlaA and MalT (panel A), and binary alignment of TMS7 region (panel B) of UlaA (sequence A) and MalT (sequence B).
Because an excellent alignment had already been published by McCoy, Ming Zhou and co-workers although it was missing the considerably sequence divergent UlaA family, UlaA family sequences were aligned in the original UlaA paper (Fig S1 in Luo *et al*. 2015), showing excellent conservation of the G58 and G286 residues in closely related species, and in VpeC. According to TCDB's (Transporter Classification Database's) strict statistical homology criteria for transporter sequence homology, the families of UlaA and ChbC/MalT are placed in different groups. It is difficult to align these families, and there is a striking length difference between the TMS7s, and in ChbC/MalT the comparatively short helix is more flanked than interpenetrated by glycine.
Using GSAT (Global Sequence Alignment Tool, developed by Vamsee Reddy at Transporter Classification Database) to detect hyper-remote homology using the so called "Super Family Principle" ("if A is similar to B, and B is similar to C, C must be homologous to A"), a challenging alignment of the TMS7 region of UlaA and MalT was attempted (panel B). We used 200 shuffles, a gap opening penalty of 8 and an extension penalty of 2. Multiple GSAT runs consistently identified the same conserved local region containing YXXXTG.
The first glycine we are studying in UlaA and two flanking residues are color-coded blue in panel A. The same color-coding is used for the second V-motif glycine (blue). TMS7 in UlaA is comparatively long, and the starting and ending triplets are color-coded yellow in panel A. For MalT, the region from approximately TMS4 that was considered in the original conservation study is color-coded red. The TMS7 of MalT is color-coded green, and may include the segment color-coded turquoise. Although the alignment is poor, there is a motif identified in GSAT (highlighted in pea green).

Figure 8. Nearby (5 Å) atoms of CA of G286 of UlaA (4RP9).
For the nearby atoms study, we took the file 4RP9 and extracted the ATOM lines only in a Linux environment. We considered ATOM CA (#4421) of residue 286 (glycine, located at 4.531; 55.238; 58.715). There is a total of 7044 atoms. Using a Perl script and the distance formula, we found nearby atoms. The residues with nearby atoms are: 51, 54, 55, 282, 283, 284, 285, 286(*=green), 287, 288, 289 and 290. Outputting these atoms in a new PDB made it clear there is room for a side-chain.

Figure 9, A–D (cartoonwise). Triplicate 1 (“Rep 1”) of MD simulation of MalT substrate release by TMS7 mechanism (outward open, substrate present), RR distance map comparing timestep 5 ns and 10 ns simulation time (a), timestep 10 ns and 15 ns simulation time (b), timestep 15 ns and 20 ns simulation time (c), timestep 20 ns and 25 ns simulation time (d).

Figure 10, A–D. Triplicate 2 (“Rep 2”) of MD simulation of MalT substrate release by TMS7 mechanism (outward open, substrate present), RR distance map comparing timestep 5 ns and 10 ns simulation time (a), timestep 10 ns and 15 ns simulation time (b), timestep 15 ns and 20 ns simulation time (c), timestep 20 ns and 25 ns simulation time (d).

Figure 11, A–D. Triplicate 3 (“Rep 3”) of MD simulation of MalT substrate release by TMS7 mechanism (outward open, substrate present), RR distance map comparing timestep 5 ns and 10 ns simulation time (a), timestep 10 ns and 15 ns simulation time (b), timestep 15 ns and 20 ns simulation time (c), timestep 20 ns and 25 ns simulation time (d).

Fig. 12, A – C. Δ-distance maps representing the difference between the outward open C2A and $P2_1A$ crystal forms of UlaA.

(A) Bypassing the “inward-outward” convention established earlier, we determined the PDB: 4RP8.A-4RP9 distance map. As above, blue indicates a positive number, red, a negative number. Hence, blue coloring means the distance in the $P2_1A$ state of UlaA (PDB: 4RP8.A) is greater than in the C2A conformation of UlaA (PDB: 4RP9), or that the corresponding atomic distance increased. For example, the distance between the N-terminal ends of TMS2 and TMS4 increased, including the distance between Ile42 (atom# CD1) and Gln140 (atom# OE1), which increased from 33.4 A (4RP9) to 34.0 A (4RP8) (hence colored blue). Conversely, the distance between mid-TMS6 and a loop between TMSs 8-9 (including atoms #CB of Ile242 CB & CD2 of Leu352) decreased from >27.4 A (4RP9) to <27.4 A (4RP8) (hence colored red). The color scale used goes from approximately -1.5 to +1.5 A, but most cells have absolute values that are smaller than ±0.5 A. The two horizontal arrows indicate the positions of the pivotal residues Gly58 and Gly286. Gly58 appears to be associated with a 7 residues-wide stationary region, possibly associated with the need to uncouple TMS2 movement from mid TMS2 residues interacting with the substrate. (B) ESCET 0.7 was used to normalize the diagram. What appears red and blue in the normalized diagram is >300 σ more extreme than the background, indicating that the differences are greater than the resolution limit of both structures. (C) RR distance maps in Chimera 1.10 were used to determine that the “red cross” reached 0.85 S.D. but that the two “blue crosses” were closer to 0.5 S.D.

2˚ element (UlaA)	3˚ element	2˚ element (ChbC)	3˚ element
**AH1**	V motif 1	AH1	V motif 1
**TMS1**	V motif 1	TMS1	V motif 1
		Helical segment*	V motif 1
**TMS2**	V motif 1	TMS2 (a/b)**	V motif 1
		TMS3	First core
**HP1b (β1)**	First core	BH x 3 ***	First core
**HP1a**	First core		
**TMS3**	First core		
**TMS4b (β2)**	First core	TMS4	First core
**TMS4a (partial)**	First core		
**TMS5**	First core	TMS5 ****	First core
**HP2b (β3)**	First core		
**HP2a**	First core		
**AH2**	V motif 2	AH2	V motif 2
**TMS6**	V motif 2	TMS6	V motif 2
**AH3 (fragment)**	V motif 2		
**TMS7**	V motif 2	TMS7	V motif 2
**HP3b (β4)**	Second core	HP1a*	Second core
**HP3a**	Second core	HP1b	Second core
**TMS8**	Second core	TMS8a, b*****	Second core
**TMS9b (β5)**	Second core	TMS9	Second core
**TMS9a**	Second core	TMS9	Second core
**TMS10**	Second core	TMS10	Second core
**HP4b (β6)**	Second core	Coil, before HP2	Second core
**HP4a**	Second core	HP2	Second core
**TMS11 (extra)**	Extra, terminal		

Table 1. Tabular form of a proposed alignment of secondary structural elements of UlaA and ChbC. This alignment is not "linear" since we cannot rearrange sequential elements.

*) TMS2 is tri-fragmented in ChbC, but continuous in UlaA.

**) Three β-strands and a tiny helical segment are placed internally to the core TMS3 (essentially analogous to the placement of HP1a and HP1b between TMSs 8 and 9 of ChbC). This could be envisaged by a segmental duplication involving the β-strands, giving rise to the parallel orientation, but this is in principle the same as the strand followed by the helical hairpin structure in UlaA.

***) The N-terminal part of TMS5 is fragmented, likely to be an evolutionary precursor of the arrangement in the second core domain, where HP2 has been tilted back into the membrane.

****) In ChbC, the hairpin elements are intertwined between the core TMSs of the second core, while in UlaA, they are placed on either side of the core 3-TMS group.

*****) In ChbC, TMS8 is split in two halves, whereas in UlaA, the adjacent TMS9 is split.

Table 2. Hydrogen bond distances in the C2A state of the Vitamin C binding site.

Donor	Hydrogen	Acceptor	D..A distance	D-H..A distance
THR 86.A OG1	THR 86.A HG1	ASC 501.A O2 *	3.008	2.320 ‡
TYR 87.A OH	TYR 87.A HH	ASC 501.A O3	2.571	1.775 ‡
HIS 135.A NE2	HIS 135.A HE2	ASC 501.A O5	2.998	2.183
GLN 139.A NE2	GLN 139.A HE22	ASC 501.A O2 *	2.927	2.219
GLN 195.A NE2	GLN 195.A HE21	ASC 501.A O1	2.992	2.141
ASC 501.A O2 **	no hydrogen	THR 86.A OG1	3.008	N/A
ASC 501.A O2 **	no hydrogen	TYR 87.A OH	3.479	N/A ‡
ASC 501.A O2 **	no hydrogen	GLN 139.A OE1	2.748	N/A ‡
ASC 501.A O3	no hydrogen	TYR 87.A OH	2.571	N/A

*,** = examples of hydrogen bonds that are competing, redundant or incompatible.
‡ = not listed in Table 3; this bond was likely broken.

Table 3. Hydrogen bond distances in the $P2_1A$ state of the Vitamin C binding site.

Donor	Hydrogen	Acceptor	D..A distance	D-H..A distance
HIS 135.A NE2	HIS 135.A HE2	ASC 501.A O5	2.948 (2.492**)	2.004*
GLN 139.A NE2	GLN 139.A HE22	ASC 501.A O2	2.710 (2.903**)	1.800*
GLN 195.A NE2	GLN 195.A HE21	ASC 501.A O1	3.075 (2.972**)	2.078*
ASC 501.A O2	ASC 501.A H2	THR 86.A OG1	3.008 (2.830**)	2.057†
ASC 501.A O3	ASC 501.A H3	TYR 87.A OH	2.483 (2.875**)	1.651*
ASC 501.A O5	ASC 501.A HO5	HIS 194.A NE2	2.894 (3.029**)	1.951*

*=measurement was reduced and closer to the typical optimal D-H..A length (~1.9).
**=D..A distance from SHELXL file with hydrogen atoms.
†=the D..A distance measurement was unaffected; the D-H.AA distance measurement was unavailable in the C2A open state (see Table 2).
